# Short-Term Effects of Focal Muscle Vibration on Motor Recovery After Acute Stroke: A Pilot Randomized Sham-Controlled Study

**DOI:** 10.3389/fneur.2019.00115

**Published:** 2019-02-19

**Authors:** Massimiliano Toscano, Claudia Celletti, Alessandro Viganò, Alberto Altarocca, Giada Giuliani, Tommaso B. Jannini, Giulio Mastria, Marco Ruggiero, Ilaria Maestrini, Edoardo Vicenzini, Marta Altieri, Filippo Camerota, Vittorio Di Piero

**Affiliations:** ^1^Department of Human Neurosciences, Sapienza University of Rome, Rome, Italy; ^2^Department of Neurology, Fatebenefratelli Hospital, Rome, Italy; ^3^Physical Medicine and Rehabilitation Division, Umberto I University Hospital, Rome, Italy; ^4^Department of Anatomy, Histology, Forensic Medicine and Orthopaedics, Sapienza University of Rome, Rome, Italy

**Keywords:** stroke, acute stroke, focal muscle vibration, motor recovery, stroke rehabilitation, neural plasticity

## Abstract

Repetitive focal muscle vibration (rMV) is known to promote neural plasticity and long-lasting motor recovery in chronic stroke patients. Those structural and functional changes within the motor network underlying motor recovery occur in the very first hours after stroke. Nonetheless, to our knowledge, no rMV-based studies have been carried out in acute stroke patients so far, and the clinical benefit of rMV in this phase of stroke is yet to be determined. The aim of this randomized double-blind sham-controlled study is to investigate the short-term effect of rMV on motor recovery in acute stroke patients. Out of 22 acute stroke patients, 10 were treated with the rMV (vibration group–VG), while 12 underwent the sham treatment (control group–CG). Both treatments were carried out for 3 consecutive days, starting within 72 h of stroke onset; each daily session consisted of three 10-min treatments (for each treated limb), interspersed with a 1-min interval. rMV was delivered using a specific device (Cro®System, NEMOCO srl, Italy). The transducer was applied perpendicular to the target muscle's belly, near its distal tendon insertion, generating a 0.2–0.5 mm peak-to-peak sinusoidal displacement at a frequency of 100 Hz. All participants also underwent a daily standard rehabilitation program. The study protocol underwent local ethics committee approval (ClinicalTrial.gov NCT03697525) and written informed consent was obtained from all of the participants. With regard to the different pre-treatment clinical statuses, VG patients showed significant clinical improvement with respect to CG-treated patients among the NIHSS (*p* < 0.001), Fugl-Meyer (*p* = 0.001), and Motricity Index (*p* < 0.001) scores. In addition, when the upper and lower limb scales scores were compared between the two groups, VG patients were found to have a better clinical improvement at all the clinical end points. This study provides the first evidence that rMV is able to improve the motor outcome in a cohort of acute stroke patients, regardless of the pretreatment clinical status. Being a safe and well-tolerated intervention, which is easy to perform at the bedside, rMV may represent a valid complementary non-pharmacological therapy to promote motor recovery in acute stroke patients.

## Introduction

Stroke is the leading cause of long-term disability (1), mostly because of incomplete functional recovery post-stroke with more than half of stroke survivors aged 65 and over exhibiting reduced mobility (2).

Furthermore, it remains unclear which is the most effective training protocol for rehabilitation of a paretic limb, as do the factors underlying recovery of motor function. A growing body of evidence from neuroimaging (3) and neurophysiological studies (4) indicate that a focal brain lesion resulting from stroke may trigger structural and functional changes in perilesional and remote brain regions. In fact, a stroke lesion can directly damage the motor pathways as well as alter the balance of excitatory and inhibitory influences within the motor network, both in the affected and unaffected hemisphere. Therefore, a modulation of this network, by acting on brain plasticity and network relearning, may be crucial for the recovery of motor function after stroke.

From this point of view, one of the most effective modulators of cortical motor and somatosensory structures is repeated sensory input (5). Muscle vibration is a strong proprioceptive stimulus, which, at low amplitudes, preferentially produces Ia fiber afferent input and reaches both the SI and M1 directly. The specific pattern of direct connections linking SI and M1 cortices may provide the anatomical substrate for the role muscle vibration plays in reorganizing the motor and somatosensory cortices (6–9).

In particular, a repetitive focal muscle vibration (rMV) at a fixed low frequency of 100 Hz rMV, applied during a voluntary contraction, may induce both prolonged changes in the excitatory/inhibitory state of the primary motor cortex in healthy subjects (10), and long-term changes of motor performance in patients as well (11).

A recent study using transcranial magnetic stimulation showed that rMV therapy, combined with physiotherapy, helped to reduce abnormalities of both the corticospinal excitability and the intracortical inhibitory systems in the damaged hemisphere of chronic stroke patients (12). Interestingly, the clinical and neurophysiological changes lasted for at least 2 weeks after the end of rMV treatment and were related to a decrease in spasticity and increase in motor function. In chronic stroke patients, two different studies demonstrated that rMV treatment may improve the functional ability of the upper (13) and lower limb (14).

The structural and functional changes within the motor network that underlie motor recovery occur in the immediate few hours after stroke; thus, it seems to be crucial to understand if it is possible to act on them during the acute phase of stroke, in order to improve stroke rehabilitation. Very few studies have been carried out on acute stroke patients so far, and none of those used rMV in the acute stage of stroke.

The aim of the present randomized double-blind sham-controlled study is to investigate the effects of rMV on motor recovery in acute stroke patients.

## Materials and Methods

### Subjects

We prospectively examined consecutive patients admitted to our Stroke Unit for ischemic or hemorrhagic stroke within 72 h from symptom onset. Inclusion criteria were: age>18, first ever stroke detected by Magnetic Resonance Imaging (MRI) or Computer Tomography (CT) scan, motor deficit of the upper and/or lower limb; ability to perform at least a minimal isometric voluntary contraction of the affected limb. We excluded patients with TIA, or rapidly improving stroke, cerebral venous thrombosis or presenting with aphasia, neglect, or apraxia. Those patients who were on drugs active at the central nervous system level at the time of the recruitment have been excluded as well.

The study protocol underwent local ethics committee approval (“Policlinico Umberto I of Rome” Ethics committee); the clinical trial was registered in the ClinicalTrial.gov database (NCT03697525). Written informed consent was obtained from all of the participants. The study was conducted in conformity with the ethical standard, according to the Declaration of Helsinki.

### Experimental Design

**Figure 1:**
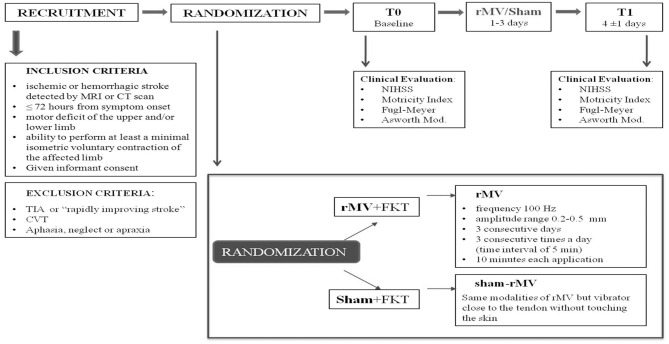
Study flow chart.

This is a prospective randomized double-blind sham-controlled study. After enrollment (T-0), patients were randomly placed into the vibration group (VG) or the control group (CG), by using a computer-generated randomization list. VG patients received rMV treatment while those of CG received the sham one. Both treatments were carried out during the 1st, 2nd, and 3rd day after enrollment. Physio kinesitherapy (PT) was carried out in all patients every day, starting soon after T-0 clinical evaluation. Patients were re-evaluated after 4 ± 1 days (T-1), at the end of treatment (see Figure 1 for the study flow chart).

### Clinical Evaluation

Upon admission, all participants' demographic details and medical history were recorded. All patients underwent a clinical examination, performed at all time-points by an experienced investigator, blinded to the group assignment and different from the recruiting one. Clinical evaluation consisted of stroke severity evaluation, by means of NIH Stroke Scale (15); motor and functional limbs abilities were evaluated by using both the Fugl-Meyer scale (16–18), and the Motricity Index (19); spasticity was assessed with Ashworth scale, modified by Bohannon and Smith (20).

### Physiotherapy (PT)

All participants underwent a 1-h daily rehabilitation session (for each treated limb), which included passive/active movements, mobilization, and proprioceptive neuromuscular facilitation of the affected limb.

Before treatment, the physical therapist was instructed about duration, frequency, and content of therapy in order to ensure uniformity in treatment procedures and blinded to patients' treatment allocation.

### Repetitive Focal Muscle Vibration (rMV)

rMV was delivered using a specific device that consisted of an electromechanical transducer, a mechanical support, and an electronic control device (Cro®System, NEMOCO srl, Italy). A mechanical arm permitted the transducer to be placed on the treatment site and to deliver the vibration at bedside, with patients placed supine; the support was rigidly anchored to the floor to guarantee good mechanical contact with tissue.

The transducer was applied perpendicular to the target muscle's belly (flexor carpi radialis and the biceps brachii for the upper limb, and/or over the rectus femoris for the lower limb treatment), near its distal tendon insertion. It generated a sinusoidal displacement of 0.2–0.5 mm (peak to peak); this parameter were used since small vibration amplitudes are effective for stimulating Ia afferents and for avoiding tonic vibration reflex as well (21, 22). Considering that Ia afferents can fire synchronously with vibration frequencies up to 80–120 Hz (23, 24), vibration characteristics were set to 100 Hz.

The rMV treatment was delivered for 3 consecutive days by two trained physiatrists; each daily session consisted of three 10-min vibration treatment (for each treated limb), separated with a 1-min interval. Otherwise, sham rMV was carried by positioning the vibrator close to the tendon but without touching the skin. In this condition, patients were only subject to the faint buzzing sound of the vibrator (13). In those patients who had a motor deficit of both the upper and the lower limb, the interventions (i.e., rMV and sham) were applied separately and in succession (1-min interval) to both limbs.

To increase response to vibration, during both the treatments (i.e., rMV and sham), patients were required to make a mild voluntary contraction (22, 25) of the treated muscle. On the other hand, during the intervals, patients were asked to relax the muscle.

### Statistical Analysis

We assessed the normality of the distributions with the Shapiro-Wilk Normality Test. According to the result of normality analysis, Student's *T*-test for paired samples or Wilcoxon test for paired samples were used to analyze clinical and neuroradiological difference between the two groups (i.e., VG e CG).

To investigate differences over time (from T-0 to T-1) between the two groups concerning clinical end-points (i.e. NIHSS, Fugl-Meyer, Motricity Index, and Ashworth scales score), we adopted two different analyses: the analysis of variance (ANOVA) allowed to compare the two groups in terms of clinical improvement expressed as difference between T-1 and T-0 scales score (ΔT-1-T-0). Moreover, by means of the analysis of variance for repeated measures (ANOVA-RM) with Tukey *post-hoc* analysis, we also analyzed clinical improvement expressed as over time repeated measures.

The *P*-value level of significance throughout the statistical analysis was set at 0.05, considering Bonferroni correction. Statistical analysis was conducted with the SPSS software package for Windows, release 22.0.

## Results

We recruited 22 patients (14 males, mean age 67 ± 13 years) in the acute phase of stroke (mean time from stroke: 43.9 ± 18.9 h). All patients were right-handed. None of them were treated with mechanical thrombectomy nor received any thrombolytic treatment. Twelve patients were treated with antiplatelet agents. None of the patients had sensory deficit as assessed by the NIHSS.

**Figure 2:**
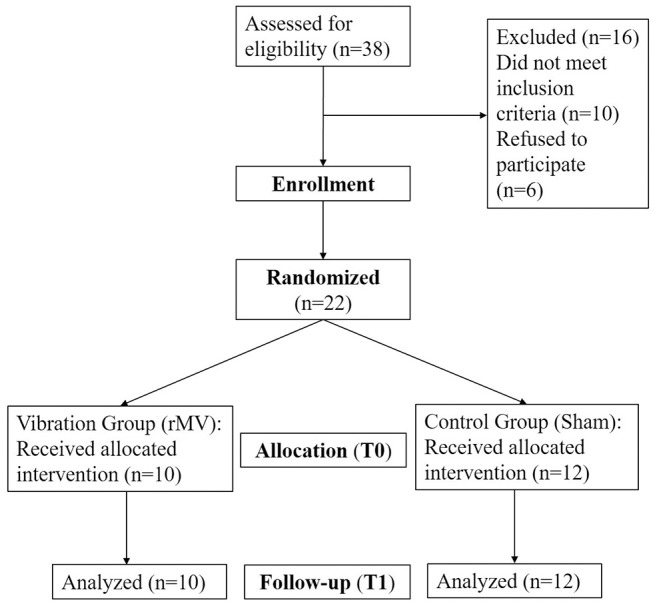
Diagram showing the flow of participants.

After the randomization, 10 patients were treated with the rMV (VG), while 12 underwent the sham treatment (CG) (see Figure 2 for the diagram showing the flow of participants). None of the treated patients complained side effects during (e.g., pain) or after the vibration treatment.

Two patients (1 VG, 1 CG) were treated only on the upper limb, 4 patients (2 VG, 2 CG) only on the lower one, and the remaining 16 patients (6 VG, 8 CG) on both the limbs.

Differences between VG and CG in term of demographic data, stroke characteristics and clinical features are shown in Table 1.

**Table 1:** Univariate Analysis: significant demographic data, medical history, clinical and neuro-anatomical characteristics according to the type of treatment.

		**rMV**	**SHAM**	***p*-value**
		***N* = 10 *n*(%)**	***N* = 12 *n*(%)**	
Age	(Mean ±*SD*)	64.70 ± 17.24	69.50 ± 7.3	0.39
Sex	Male	8 (80)	6 (50)	0.16
	Female	2 (20)	6 (50)	
Time from stroke	(Hours)	45 ± 20.4	43 ± 18.4	0.81
Stroke Type	Ischemic	4 (40)	8 (66.7)	0.39
	Hemorrhagic	4 (40)	2 (16.7)	
	Both	2 (20)	2 (16.7)	
Stroke	Cortical	3 (30)	4 (33.3)	0.80
Localization	Subcortical	4 (40)	4 (33.3)	
	Brainstem	1 (10)	0	
	Cortico-subcortical	2 (20)	4 (33.3)	
Stroke Side	Right	6 (60)	4 (33.3)	0.23
	Left	4 (40)	8 (66.7)	
	Bilateral	0	0	
CAD (Coronary Artery disease)		7 (70)	10 (83.3)	0.13
Smoke		2 (20)	6 (50)	0.16
Hypertension		8 (80)	8 (66.7)	0.51
Diabetes		2 (20)	4 (33.3)	0.51
Hypercholesterolemia		4 (40)	6 (50)	0.66
Atrial Fibrillation		2 (20)	0	0.11
Previous	No	8 (80)	8 (66.7)	0.89
Stroke	Ischemic	1 (10)	4 (33.3)	
	Hemorrhagic	1 (10)	0	
Cardiac Failure		1 (10)	0	0.28
NIHSS (T0)	(Mean ±*SD*)	12.4 ± 4.09	10 ± 3.22	0.13

The two groups of stroke patients did not differ for age (*p* = 0.39), sex (*p* = 0.16), stroke type (*p* = 0.39), lesion side (*p* = 0.23), stroke localization (*p* = 0.23), and for the presence of major cerebrovascular risk factors. Univariate analysis did neither show any difference between the two groups regarding both the stroke severity upon admission, (NIHSS score–VG: 12.4 ± 4.09; CG: 10±3.22; *p* = 0.13), and the mean time between rMV treatment and stroke (VG: 45 ± 20.4 h; CG 43±18.4 h; *p* = 0.8).

**Figure 3:**
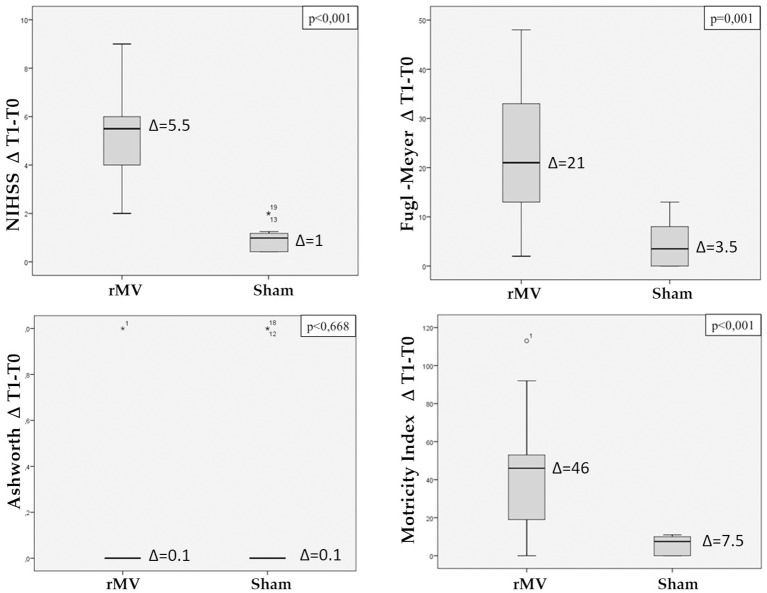
Box plot with Interquartile Range (IQR) distribution of the difference between T1 and T0 scales values (NIHSS, total Fugl-Meyer, total Motricity Index, Ashworth modified) in patients treated with rMV and in those treated with sham-rMV. ANOVA's *p*-value for comparison of the variable between the two groups is reported on the top of each the figure.

Analysis of variance (ANOVA) showing difference between T-1 and T-0 scores (ΔT1-T0) for each clinical variable (i.e., NIHSS, Fugl-Meyer, Motricity Index e Ashworth Modified) is reported in Figure 3. Patients treated with rMV (VG) had a significant clinical improvement with respect to those treated with a sham-rMV among the NIHSS (*p* < 0.001), Fugl-Meyer (*p* = 0.001), and Motricity Index (*p* < 0.001) scores.

Only five patients (3 VG, 2 CG) had post-stroke spasticity, with a maximum modified Ashworth scale (MAS) score of 1 (i.e., very slight increase in the muscle tone); no difference in the MAS score were found between groups (*p* = 0.668).

**Figure 4:**
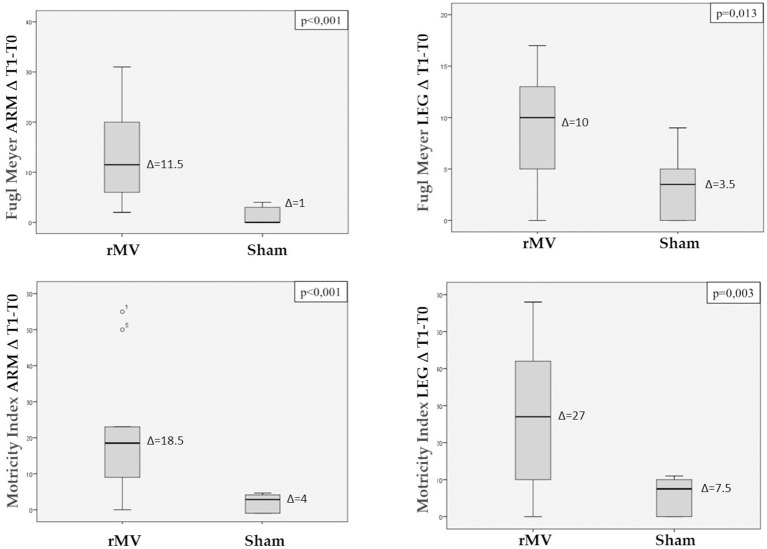
Box plot with Interquartile Range (IQR) distribution of the difference between T1 and T0 scales values (Fugl-Meyer arm, Fugl-Meyer leg, Motricity Index arm and Motricity Index leg) in patients treated with rMV and in those treated with sham-rMV. ANOVA's *p*-value for comparison of the variable between the two groups is reported on the top of each the figure.

By comparing the Fugl-Meyer and Motricity Index scales scores separately for the upper and the lower limb, VG patients were found to have a better clinical improvement at all the clinical end points (Arm: Fugl-Meyer *p* < 0.001, Motricity Index *p* < 0.001; Leg: Fugl-Meyer *p* = 0.013, Motricity Index *p* < 0.001) (Figure 4).

**Figure 5:**
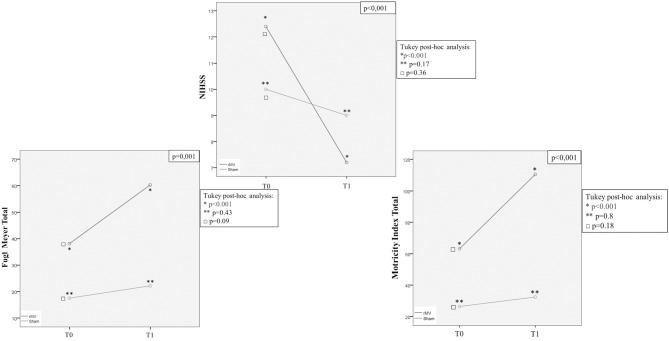
ANOVA for Repeated Measures (ANOVA-RM) with Tukey *post-hoc* analysis: T0 and T1 mean values (NIHSS, total Fugl-Meyer, total Motricity Index, Ashworth modified) in patients treated with rMV (blue line) and in those treated with sham-rMV (green line).

**Figure 6:**
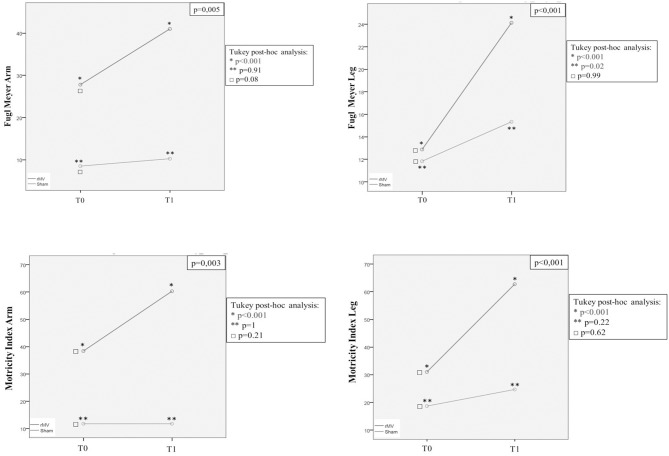
ANOVA for Repeated Measures (ANOVA-RM) with Tukey *post-hoc* analysis: T0 and T1 mean values (Fugl-Meyer arm, Fugl-Meyer leg, Motricity Index arm and Motricity Index leg) in patients treated with rMV (blue line) and in those treated with sham-rMV (green line).

Analysis of variance for repeated measures (ANOVA-RM) with Tukey *post-hoc* analysis, allowed us to analyze the clinical improvement expressed as over time repeated measures for each clinical end-point (Figures 5, 6). VG patients showed a better clinical improvement with respect to CG patients in terms of stroke severity assessed by NIHSS (*p* < 0.001), and of Fugl-Meyer (*p* = 0.001) and Motricity Index scale score (*p* < 0.001). The better motor outcome of the rMV-treated patients was confirmed for the upper and the lower limb, separately (Arm: Fugl-Meyer *p* = 0.005, Motricity Index *p* = 0.003; Leg: Fugl-Meyer *p* < 0.001, Motricity Index *p* < 0.001).

Tukey *post-hoc* analysis showed that ANOVA-RM significance was only due to rMV patients clinical improvement from T-0 to T-1 (rMV T-0-T-1: NIHSS *p* < 0.001; Fugl-Meyer tot *p* < 0.001; Fugl-Meyer Arm *p* < 0.001; Fugl-Meyer Leg *p* < 0,001; Motricity Index tot *p* < 0.001; Motricity Index Arm *p* < 0.001; Motricity Index Leg *p* < 0.001), but the Fugl-Meyer Arm (sham-rMV T0-T1: Fugl-Meyer Arm *p* = 0.02). In fact, this analysis did not show any difference between groups regarding the pre-treatment clinical status (NIHSS: *p* = 0.36; Fugl-Meyer Tot: *p* = 0.09; Fugl-Meyer Arm: *p* = 0.08; Fugl-Meyer Leg: *p* = 0.99; Motricity Index Tot: *p* = 0.18; Motricity Index Arm: *p* = 0.21; Motricity Index Leg: *p* = 0.62).

## Discussion

Focal repetitive muscle vibration (rMV) is a safe and well-tolerated intervention which is easy to perform at the bedside, and promotes neural plasticity and long-lasting motor recovery in chronic stroke patients (12).

Although much evidence exists of the efficacy of focal muscle vibration in the chronic phase, the clinical benefit in the very acute phase of stroke is yet to be determined. From a clinical point of view, the reason why this issue is crucial, is that those structural and functional changes within the motor network that underlie the motor recovery after stroke occur in the very first hours after stroke. To our knowledge, no studies have been carried out to investigate the effect of rMV on motor recovery in the acute phase of stroke so far.

Our data show that the rMV intervention can consistently improve motor outcome in a cohort of acute stroke patients. In fact, patients with stroke treated with rMV (VG) had a significant clinical improvement compared to those treated with a sham-rMV as shown by improved NIHSS (*p* < 0.001), Fugl-Meyer (*p* = 0.001), and Motricity Index (*p* < 0.001) scores, regardless the different baseline clinical status, or the different stroke characteristics (stroke type, side or localization of stroke lesion and so on).

The neural substrates underlying motor recovery in the acute phase of stroke are still a matter of debate. Despite the role of the hyperactivation of several cortical areas in both the affected and in the unaffected hemisphere being still unclear, ipsilesional M1 is widely thought to represent the most effective target for rehabilitation therapy (26, 27). This has become a milestone since pioneering studies described how the integrity and or over-activation of the lesioned hemisphere's motor cortex (ipsilesional M1) related to better post-stroke motor recovery (28–30).

Thus, in our opinion, the primary mechanism by which rMV may improve motor recovery after acute stroke is through a direct action on the ipsilesional motor cortex. In detail, the repeated muscle vibration produces a repeated sensory input that reaches M1 directly, via Ia fiber afferent input (6–9), thereby leading to an improvement of functional ability of the affected limb by means of an intrinsic plasticity-related mechanism (11, 13).

An additional mechanism that may be involved in the rMV-induced motor recovery in acute stroke, probably concurrent with the direct action on ipsilesional M1, entails the changes in perilesional brain regions triggered by the focal brain lesion and their connections to the spinal cord motor neurons.

The recruitment of secondary brain structures, due to the capability to establish and consolidate new neural networks in response to a change in the environment (i.e., neuroanatomical plasticity), has been described in the acute phase, especially in those patients with greater motor impairment. This compensative recruitment (i.e., increased activity) is not “maladaptive” because the effects of TMS disruption have demonstrated that their activity is functionally significant (31); nevertheless, it leads to an incomplete recovery (32). The main reason is that the projections from ipsilateral non-primary motor areas to spinal cord motor neurons are less numerous and less efficient at exciting spinal cord motor neurons than those from M1 (30, 33, 34).

Considering that the focal muscle vibration represents a strong proprioceptive stimulus which is able to produce substantial neurophysiological changes also at a peripheral level, it is probably also able to induce synaptic plasticity at the Ia-motoneuron synapse level, thereby increasing the effectiveness of these cortical-spinal connections. In light of this, it is intriguing that a recent study reported that rMV was able to induce long-term depression-like plasticity in specific spinal cord circuits, depending on the muscle vibrated (22).

Thus, our hypothesis is that rMV could drive motor recovery by also acting on spinal cord plasticity, namely by making the projections from secondary motor areas to spinal motor neurons more active and efficient. This mechanism could be of particular relevance in patients with higher motor impairment. Moreover, considering that the secondary motor areas (e.g., PMd) have prominent bilateral connections to the spinal cord (32), one might speculate that this mechanism is able also to act on interhemispheric imbalance involving hyperexcitability of the contralesional hemisphere, whose modulation may have a pivotal, although still unclear, role in motor recovery after stroke (27, 33).

Finally, a possible role of rMV in reducing spasticity when applied to the spastic muscles of hemiplegic limbs in post-stroke patients as also been suggested (13, 35, 36). Among the whole population of recruited patients, we found a mild increase in muscle tone in 5 patients, with no difference between the two groups in Ashworth modified score changes. A possible explanation of this datum is that we evaluated stroke patients in the very acute phase of stroke, whereas spasticity usually develops after several weeks after stroke. Moreover, the very slight increase (with a maximum MAS score of 1) probably did not allow finding a statistical difference between groups. Anyway, there are evidences of spasticity development in the early time course of stroke (37). It would be therefore intriguing to perform a follow-up study to investigate whether this datum is merely due to the timing of spasticity assessment, or if we somehow were able to prevent the spasticity by stimulating the proprioceptive system since the very acute phase (38).

A limitation of the study is that, due to the peculiar emergency setting of the acute Stroke Unit, patients were asked to perform a mild voluntary contraction without measurement of the performed contraction with visual EMG feedback. Moreover, due to the relatively low number of patients, we were not able to perform a multivariate analysis to avoid all stroke-related clinical bias. That notwithstanding, to have further evidences of the role of an intrinsic mechanism more than one linked to patients' clinical characteristic (as already demonstrated in the chronic phase), we evaluated motor outcome by separately analyzing the ΔT1-T0 Fugl-Meyer and Motricity Index scales scores of the upper limb and those of the lower limb. Also, in this, case SG patients were found to have a better clinical improvement at all the clinical end-points (Arm: Fugl-Meyer *p* < 0,001, Motricity Index *p* < 0.001; Leg: Fugl-Meyer *p* = 0.013, Motricity Index *p* < 0.001).

With the same goal in mind, we also analyzed clinical improvement expressed as over-time repeated measures by means of the analysis of variance for repeated measures (ANOVARM) with Tukey *post-hoc* analysis. We found that, for all the clinical end-points analyzed except Fugl-Meyer Arm, the significance of patients' clinical improvement from T0 to T1 was exclusively due to rMV treatment (rMV T0-T1: NIHSS *p* < 0.001; Fugl-Meyer Tot *p* < 0.001; Fugl-Meyer Arm *p* < 0.001; Fugl-Meyer Leg *p* < 0.001; Motricity Index Tot *p* < 0.001; Motricity Index Arm *p* < 0.001; Motricity Index Leg *p* < 0.001); this is important because a minimal improvement is somehow expected because of the PT treatment and because of the stroke natural clinical history as well. Moreover, also when expressed as over-time repeated measures, VG better clinical outcome was independent from the different initial clinical status; interestingly, rMV-related recovery was even more consistent in patients with a more severe stroke in terms of NIHSS, which supports the hypothesis of a plasticity-based intrinsic mechanism being responsible for the better motor recovery of stroke patients treated with rMV in the acute phase of stroke.

However, addressing the plasticity-based mechanisms underlying the rMV-induced motor recovery after stroke does however, go beyond the main clinical purpose of our study. Thus, further RCTs are needed to draw conclusions on this specific issue.

Regarding the main outcome of our study, our data provides the first evidence that the rMV intervention can improve motor outcome in a cohort of stroke patients regardless the different baseline clinical status, or the different stroke characteristics.

## Conclusions

This study provided the first evidence that repetitive focal muscle vibration (rMV), when combined with physiotherapy, is able to improve motor outcome in a cohort of stroke patients, even when performed in the very acute phase of stroke. As a safe and well-tolerated intervention, which is easy to perform at bedside, rMV may represent a valid complementary non-pharmacological therapy to promote motor recovery in acute stroke patients.

## Author Contributions

MT: study design, manuscript preparation, data collection, statistical analysis, blinding overview; CC: rMV execution, manuscript preparation; AV: clinical evaluation, manuscript preparation; AA: clinical scales' implementation and review; GG and TJ: recruitment, demographic details, and medical history record; GM and MR: literature review, ethics committee; IM: literature review, manuscript review; EV and MA: data interpretation, manuscript review; FC: rMV execution, manuscript review, physio kinesitherapy overview; VDP: data interpretation, manuscript review, coordination between specialists.

### Conflict of Interest Statement

The authors declare that the research was conducted in the absence of any commercial or financial relationships that could be construed as a potential conflict of interest.
